# Dysnatremias in Chronic Kidney Disease: Pathophysiology, Manifestations, and Treatment

**DOI:** 10.3389/fmed.2021.769287

**Published:** 2021-12-06

**Authors:** Soraya Arzhan, Susie Q. Lew, Todd S. Ing, Antonios H. Tzamaloukas, Mark L. Unruh

**Affiliations:** ^1^Department of Internal Medicine, University of New Mexico School of Medicine, Albuquerque, NM, United States; ^2^Department of Medicine, George Washington University, Washington, DC, United States; ^3^Department of Medicine, Stritch School of Medicine, Loyola University Chicago, Maywood, IL, United States; ^4^Research Service, Raymond G. Murphy Veteran Affairs (VA) Medical Center, Albuquerque, NM, United States; ^5^Medicine Service, Division of Nephrology, Raymond G. Murphy Veteran Affairs (VA) Medical Center, Albuquerque, NM, United States

**Keywords:** dysnatremia, hyponatremia, hypernatremia, chronic kidney disease, hemodialysis, peritoneal dialysis

## Abstract

The decreased ability of the kidney to regulate water and monovalent cation excretion predisposes patients with chronic kidney disease (CKD) to dysnatremias. In this report, we describe the clinical associations and methods of management of dysnatremias in this patient population by reviewing publications on hyponatremia and hypernatremia in patients with CKD not on dialysis, and those on maintenance hemodialysis or peritoneal dialysis. The prevalence of both hyponatremia and hypernatremia has been reported to be higher in patients with CKD than in the general population. Certain features of the studies analyzed, such as variation in the cut-off values of serum sodium concentration ([Na]) that define hyponatremia or hypernatremia, create comparison difficulties. Dysnatremias in patients with CKD are associated with adverse clinical conditions and mortality. Currently, investigation and treatment of dysnatremias in patients with CKD should follow clinical judgment and the guidelines for the general population. Whether azotemia allows different rates of correction of [Na] in patients with hyponatremic CKD and the methodology and outcomes of treatment of dysnatremias by renal replacement methods require further investigation. In conclusion, dysnatremias occur frequently and are associated with various comorbidities and mortality in patients with CKD. Knowledge gaps in their treatment and prevention call for further studies.

## Introduction

Dysnatremias, defined usually as serum sodium concentration ([Na]) either below 135 mmol/L or above 145 mmol/L, represent the most frequently encountered electrolyte disorder in a variety of clinical settings (1), such as chronic kidney disease (CKD) (2, 3). The major clinical manifestations of dysnatremias result from disturbances of the intracellular volume of brain cells secondary to abnormalities in effective osmolarity (tonicity). Tonicity is the property of any fluid to reduce, not change, or increase the intracellular volume of cells bathed in it through osmotic fluid transfers (4). [Na] is the key clinical indicator of serum tonicity (4).

Hyponatremia can be associated with osmotic cell swelling, osmotic cell shrinking, or no change in the intracellular volume of cells. Hyponatremia causing osmotic swelling of cells (hypotonic or true hyponatremia) (5) is typically associated with a low serum osmolality but may be associated with normal or high serum osmolality in patients with low [Na] values and excessive loads of a solute with total body water (TBW) distribution, e.g., in patients with hyponatremic CKD and high serum urea levels. Hypertonic (or translocational) hyponatremia results from an excess of solutes with extracellular distribution, other than sodium salts, e.g., glucose or mannitol, causing osmotic exit of fluid from the intracellular compartment, hyponatremia, and elevated serum tonicity and osmolality (5). Isotonic hyponatremia with normal cell volume (pseudohyponatremia, or spurious hyponatremia, or artifactual hyponatremia) is encountered when low [Na] values are reported by methods requiring pre-measurement dilution of the serum sample, including flame emission spectrophotometry or indirect ion-specific electrode, and plasma solid content is abnormally high due to hyperlipidemia or hyperproteinemia; and [Na] measured by the direct ion-specific electrode is within the normal range (4, 5).

Dysnatremias result from a single or combined disturbances in the external balances of water, sodium, and potassium (6, 7). The kidney regulates [Na] through the excretion of sodium and potassium, and mainly through the production of dilute or concentrated urine under the direction of vasopressin (8). Abnormalities in urinary diluting or concentrating ability result in dysnatremias (8, 9). Patients with dysnatremia in the setting of CKD should be investigated for disturbances in the external balances of water, sodium, and potassium, and in urinary dilution or concentration (2, 10). This review addresses the pathophysiology, epidemiology, clinical manifestations, mortality, and management of dysnatremias in patients with CKD not on dialysis, and those on maintenance hemodialysis or peritoneal dialysis. Identification of aspects of dysnatremias in patients with CKD that need further studies will also be addressed in this review.

## Review

### Pathophysiology of Dysnatremias in Patients With CKD

Healthy human kidneys have the capacity to produce several liters of urine daily and can dilute the urine to a minimal osmolality of 50 mOsm/kg or to concentrate the urine to a maximal osmolality of 1,200 mOsm/kg in response to plasma vasopressin levels (9, 11). In CKD, anatomic derangements of the tubular and vascular structures of the diluting and concentrating nephron segments, disturbances in the interstitial hypertonicity of the renal medulla, impaired response of the principal cells of the concentrating nephron segments to vasopressin, and osmotic diuresis of the remaining functioning nephrons curtail the concentrating and diluting abilities of the kidneys (4, 9, 12).

In CKD, the impairment of the urinary concentrating ability outpaces that of the diluting ability (12, 13). Osmotic diuresis of the remaining functioning nephrons, primarily due to urea loads, plays a role in the impairment of the renal concentrating ability (14, 15). However, poor response to vasopressin of the principal epithelial cells in the concentrating segment of the nephron represents the major cause of loss of the concentrating ability. Defects in several stages of this response have been identified in studies of experimental CKD. Fine et al. (16) reported the impaired water permeability and the response of adenylate cyclase activity to vasopressin in a study of isolated perfused cortical collecting tubules from uremic rabbits (16). Teitelbaum and McGuinnes (17) found low levels of mRNA of the V2 receptor in principal cells of the inner medullary tubule of uremic rats. Suzuki et al. (18) reported decreased response of aquaporin 2 expressions in the inner medulla of uremic rats after water restriction, which caused a rise in plasma vasopressin levels with a blunted rise in urine osmolality.

Patients with early stages of CKD have the capability of excreting normal ingested loads of sodium and potassium salts and azotemic compounds in the urine, thus achieving steady states of sodium and potassium balance and serum concentrations of azotemic indices (i.e., creatinine and urea). The decreased renal concentrating ability in CKD obligates the larger volumes of urine in the early stages of CKD than in the healthy stage in order to excrete these solute loads (9). However, the renal ability to excrete water loads becomes progressively limited as the glomerular filtration rate declines and, consequently, the range of water intake that allows normal [Na] values becomes progressively narrower (9). Hyposthenuria or isosthenuria, which occurs in advanced stages of CKD, predispose to both hyponatremia and hypernatremia (2). Evaluation of dysnatremias in these patients should address gains or losses in sodium, potassium, and water through the kidneys, but also through the respiratory and gastrointestinal systems, and the skin (19).

Patients treated by maintenance hemodialysis or peritoneal dialysis have limited margins of water intake allowing a normal [Na] range. In addition, these patients may develop dysnatremias due to prescription errors. Hypernatremia has been reported both after hemodialysis sessions (20–22) and in peritoneal dialysis (23–25). Patients on peritoneal dialysis treated with icodextrin-containing dialysis fluids may develop hypertonic hyponatremia secondary to extracellular accumulation of icodextrin metabolites (26).

### Incidence and Prevalence of Dysnatremias in Patients With CKD and Those on Dialysis

Table 1 shows the incidence and prevalence of hyponatremia in the studies of the general population, CKD not on dialysis, hemodialysis, and peritoneal dialysis (10, 27–54). Table 2 shows the incidence and prevalence of hypernatremia in the same population segments (10, 28–30, 32–36, 38–40, 47, 53–55). The values for prevalence and incidence related to dysnatremias vary widely in each of the four categories of patients in Tables 1, 2 and overlap substantially among the four patient categories. The incidence of hyponatremia in a study of hospitalized patients was 9% in patients without CKD and increased progressively within CKD stages up to 18.1% in patients with CKD stage 5 (37). In another study, which compared adult (18–64 years) and elderly patients (>65 years) with and without CKD at presentation to the emergency department (39), the prevalence of hyponatremia was 2.8% in patients without CKD and 10.3% in those with CKD in the adult group and 14.8% in patients without CKD and 12.9% in those with CKD in the elderly group. In the same study, the prevalence of hypernatremia was 0.7% in patients without CKD and 2.0% in those with CKD in the adult group and 1.5% in patients without CKD, and 3.5% in those with CKD in the elderly group. All differences in prevalence were statistically significant. The findings of these two studies (37, 39) suggest that patients with CKD, at least those who are not elderly, are at higher risk for dysnatremias than those without CKD.

**Table 1:** Incidence/prevalence of hyponatremia based on the chronic kidney disease (CKD) status.

**References**	**Number of patients**	**[Na] cut-off (mmol/L)**	**Prevalence**	**Incidence**
**General population**				
Wald et al. (27)	53,236^a^^,^^b^	<138	37.9^a^, 38.2%^b^	–
Akirov et al. (28)	27,789^b^	<135	–	22.0%
Girardeau et al. (29)	45,834^b^	≤135	–	12.0%
Hu et al. (30)^*^	90,889^b^	<137	16.8%	7.8%
Al Mawed et al. (31)	2,488,437^b^	<135	14.4%	–
Lombardi et al. (32)	46,634^b^	<135	10.4%	–
Thongprayoon et al. (33)	60,944^b^	<135	34.6%	17.0%
**CKD not on dialysis**				
Kovesdy et al. (10)	655,493^a^	<136	26.0%	13.5%
Han et al. (34)^*^	2,182^a^	≤135	6.3%	–
Chiu et al. (35)^*^	2,093^a^	≤135	6.6%	–
Huang et al. (36)	45,333^a^	<136	27.0%	8.0%
Golestaneh et al. (37)	7,422^b^	<135	–	12.4%
Grangeon-Chapon et al. (38), (≥75)	279^b^	<135	29.4%	–
Imai et al. (39)^*^	4,562^b^	<135	14.8%	2.8%
(18–64 y/o)				
(≥65 y/o)	5,996^b^		12.9%	10.3%
**Hemodialysis**				
Waikar et al. (40)^*^	1,549^a^	<137	29.3%	–
Sahin et al. (41)^*^	697^a^	<135	5.9%	–
Hecking et al. (42)^*^	11,555^a^	<137	12.6%	–
Nigwekar et al. (43)^*^	6,127^a^	<135	26.8%	–
Rhee et al. (44)	27,180^a^	<138	41.6%	–
Dekker et al. (45)	8,883^a^	<135	12.7%	–
Baek et al. (46)	621^a^	<135	–	30.8%
Chiang et al. (47)^*^	62^a^	<136	45.2%	–
**Peritoneal dialysis**				
Kang et al. (48)	387^a^	<135	74.4%	–
Chang et al. (49)^*^	441^a^	<137	3.3%	–
Chen et al. (50)	318^a^	≤135	26.4%	–
Dimitriadis et al. (51)	166^a^	≤130	–	14.5%
Xu et al. (52)^*^	476^a^	≤135	10.5%	–
Yan et al. (53)^*^	60^a^	≤132	15.0%	–
Ravel et al. (54)	4,687^a^	<136	9.0%	–

*[Na], serum sodium concentration;*

* *prospective study;*

a *outpatient subjects;*

b *hospitalized subjects; ^c^location of patients not stated in the report*.

**Table 2:** Incidence/prevalence of hypernatremia based on the CKD status.

**References**	**Number of Patients**	**[Na] cut-off (mmol/L)**	**Prevalence**	**Incidence**
**General population**				
Wald et al. (27)	53,236^a^^,^^b^	>144	–	3.0%
Girardeau et al. (29)	45,834^b^	>145	1.9%	1.0%
Hu et al. (30)^*^	90,889^b^	>147	4.0%	0.9%
Lombardi et al. (32)	46,634^b^	>145	8.2%	–
Thongprayoon et al. (33)	60,944^b^	>145	8.2%	1.4%
Tsipotis et al. (55)	19,072^a^^,^^b^	>142	21.0%^a^, 25.9%^b^	21.0%^a^
**CKD not on dialysis**				
Kovesdy et al. (10)	655,493^a^	>145	7.0%	2.0%
Han et al. (34)^*^	2,182^a^	≥144	16.4%	–
Chiu et al. (35)^*^	2,093^a^	≥145	6.6%	–
Huang et al. (36)	45,333^a^	>145	6.0%	1.2%
Grangeon-Chapon et al. (38), (≥75)	279^b^	>145	24.7%	–
Imai et al. (39)^*^	4,562^b^	>145	2.0%	0.7%
(18–64 y/o)				
(≥65 y/o)	5,996^b^		3.5%	1.5%
**Hemodialysis**				
Waikar et al. (40)^*^	1,549^a^	≥142	18.9%	–
Nigwekar et al. (43)	6,127^a^	>145	1.2%	–
Rhee et al. (44)	27,180^a^	≥144	3.6%	–
Baek et al. (46)	621^a^	>145	–	3.5%
**Peritoneal dialysis**				
Ravel et al. (54)	4,687^a^	≥144	4.0%	–

*[Na], serum sodium concentration;*

* *prospective study;*

a *outpatient subjects;*

b *hospitalized subjects; ^c^location of patients not stated in the report*.

Several characteristics of the studies in Tables 1, 2 contributed both to the overlapping of the incidence and prevalence values between the categories of patients analyzed and to the wide range of these values in each category of patients. Important characteristics, which should be addressed in future studies in this area, are discussed below:

- Confounding conditions could have influenced the reported incidence and prevalence of dysnatremias. The studies of dysnatremias in the general population included varying percentages of patients with CKD. Conversely, a fraction of patients in studies involving patients with CKD also had other conditions associated with hyponatremia, e.g., congestive heart failure (10).
- The cut-off [Na] values for hyponatremia suggested by various authors ranged between ≤130 mmol/L and <138 mmol/L (Table 1), while the cut-off values for hypernatremia ranged between 142 mmol/L (55) and >147 mmol/L (Table 2). These ranges in [Na] cut-off values were responsible for the substantial differences in incidence and prevalence values.
- Many patients featured in Tables 1, 2 had diabetes mellitus, a condition that affects the calculations of incidence or prevalence of hyponatremia if [Na] values are not corrected for the degree of hyperglycemia. Hyperglycemia causes hypertonic hyponatremia. Not accounting for the degree of hyperglycemia in studies of dysnatremias, i.e., using the measured [Na], provides false information about the relationship among body sodium, potassium, and water. The corrected [Na], i.e., a predicted [Na] value after the correction of hyperglycemia, provides an appropriate estimate of this relationship (56). Katz (57) calculated theoretically a decrease in [Na] equal to 1.6 mmol/L for each 5.6 mmol/L rise in serum glucose concentration. Subsequently, the proposed range of coefficients for the calculation of the corrected [Na] was from 1.35 to 4.00 mmol/L reduction in [Na] for every 5.6 mmol/L rise in serum glucose (58). A review of this topic concluded that Katz's coefficient should be used for calculating the corrected [Na], with exceptions that make monitoring of [Na] and serum glucose during the treatment of hyperglycemia mandatory (56). The general form of the Al-Kudsi formula (59), which uses the Katz's coefficient to calculate the corrected [Na], is as follows where both sodium and glucose concentrations are in mmol/L (56):
  (1) $$\begin{array}{l} Corrected\;\left[Na\right]=Measured\;\left[Na\right]+\\ \;1.6\times \frac{Measured-Desired\;serum\;glucose}{5.6} \end{array}$$
  Several studies on dysnatremias calculated the corrected [Na] using the Al-Kudsi formula (30, 34, 36, 41–43, 46, 47, 49), and one study excluded from statistical analysis serum glucose levels >7.5 mmol/L (51). This last study was also the only one to address spurious hyponatremia by excluding [Na] values in patients with significant hyperlipidemia. The remaining studies in Tables 1, 2 either used coefficients different from Katz's coefficient to calculate the corrected [Na] or did not calculate it. The second influence of hyperglycemia, unique to diabetic patients on dialysis, who develop limited or no osmotic diuresis from glycosuria, pertains to thirst and consumption of water which is retained and reduces the value of [Na] after the correction of hyperglycemia (60). For these reasons, the incidence and prevalence of dysnatremias should be calculated separately in diabetic and non-diabetic CKD populations. Only one small study calculated the prevalence of hyponatremia in non-diabetic peritoneal dialysis patients (53). This study, which assessed changes in TBW and extracellular volume by repeated bioimpedance measurements, identified factors associated with the development of hyponatremia during peritoneal dialysis. These factors include large peritoneal ultrafiltration volumes causing excessive loss of sodium, and changes in nutritional status. Based on these findings, the authors of the study proposed a scheme for addressing hyponatremia in this patient population.

### Clinical Manifestations, Associations With Comorbidities, and Mortality of Dysnatremias in Patients With CKD

#### Hypotonic Hyponatremia

The clinical manifestations of hypotonic hyponatremia result from cerebral edema with the degree, rapidity of development, and duration of hyponatremia determining severity (5, 61). Brain cells undergo intracellular volume adaptation to hyponatremia. This adaptation determines the chronicity of hyponatremia. Within up to 7 h of hyponatremia, brain cells, mainly astrocytes which express aquaporin-4 in abundance (62), lose water to the cerebrospinal fluid through hydrostatic forces (63) and lose electrolytes, such as potassium, sodium, and chloride (5, 64). Subsequently, further intracellular brain cell volume reduction occurs through the loss of organic osmolytes, which is completed by 48 h (5). Hyponatremia lasting <48 h is considered acute, while hyponatremia of 48 h or longer is considered chronic.

Severe clinical manifestations of hyponatremia include seizures, coma, hypoxia secondary to noncardiogenic pulmonary edema and/or hypercapnic respiratory failure (65), and death from cerebral herniation; moderate manifestations include lethargy, disorientation, and confusion; and mild manifestations include fatigue, nausea, and headache (5). Even mild hyponatremia (130–135 mmol/L) is associated with attention deficits, which may require directed testing to be detected, gait disturbances, osteoporosis, and a high risk of fractures (5, 66–68). In patients with advanced CKD, the neurological manifestations of uremia can be confused with the manifestations of dysnatremias, and changes in serum urea concentration affect the treatment of dysnatremias. A paucity of studies exists regarding clinical manifestations related to dysnatremias in patients with CKD. One study reported depressed mental function in patients on peritoneal dialysis with hyponatremia (52).

Certain conditions increase the risk for hyponatremia in patients with CKD not on dialysis, and those on hemodialysis or peritoneal dialysis. These conditions include women gender (34, 36, 41, 44, 48, 54), race other than African American (34, 40, 42, 44, 54), low body weight or body mass index (34, 35, 40, 42–45, 49), diabetes mellitus (35, 40, 41, 43–45, 50, 54), and low serum albumin (10, 34, 35, 40–44, 46–50, 52, 54). In both hemodialysis and peritoneal dialysis patients, hyponatremia was found to be associated with a low residual renal function (43, 44, 47, 49, 51, 54) and excessive weight, most probably fluid, gains (40–42, 44, 45, 47, 48). Hyponatremia in CKD populations increases the risk for several adverse outcomes, such as hospitalization for infections (69), protein-energy malnutrition (70) and impaired cognitive function (71) in hemodialysis patients, and poor peritonitis outcomes (72) plus the higher incidence of new cardiovascular events in peritoneal dialysis patients (73).

Hyponatremia represents a risk for all-cause mortality in the general population (5) and in patients with CKD not on dialysis (10, 34–36), in whom it represents also a risk factor for the mortality from cardiovascular disease or malignancies (36). In hemodialysis patients, hyponatremia was found to be a risk factor for all-cause mortality (40, 42–47), for all-cause mortality only among patients with diabetes mellitus (41), and cardiovascular mortality (40). In peritoneal dialysis patients, hyponatremia was found to be a risk factor for all-cause mortality (49, 54, 72, 74). One study found no association between hyponatremia and 2-year mortality in peritoneal dialysis patients (50). The pathophysiological mechanisms by which hyponatremia increases the risk for mortality in patients with CKD are not well-understood. One study concluded that mortality in hyponatremic peritoneal dialysis patients is most probably due to co-morbidities (48).

Hospitalized patients with COVID-19 have a high prevalence of acute kidney injury with increased death risk (75) and dysnatremias (76–79). The syndrome of inappropriate antidiuretic hormone secretion (SIADH) was identified as the cause of hyponatremia in two cases of COVID-19 (80, 81). Both hyponatremia and hypernatremia represent mortality risks in patients with COVID-19 (75–79).

#### Hypernatremia

Acute hypernatremia causes lesions in the brain, such as cell shrinkage, petechial and subarachnoid hemorrhages, hematomas, subdural fluid collections, vascular congestion, and venous thrombosis (82). Children with hypernatremia develop irritability, restlessness, muscular twitching, hyperreflexia, and seizures. Elderly patients with hypernatremia rarely develop seizures, but manifest lethargy, delirium, and coma. Less frequent manifestations of hypernatremia include fever, nausea, and vomiting. Alert hypernatremic patients have intense thirst (82). Higher [Na] values are associated with the progression of established CKD independently of other risk factors (83).

Conditions presenting a risk for hypernatremia in patients with CKD not on dialysis include men's gender, older age, heart failure, low estimated glomerular filtration rate, and high levels of body mass index, systolic blood pressure, and serum albumin (34). Conditions predisposing patients on hemodialysis or peritoneal dialysis to hypernatremia will need further investigation.

Hypernatremia increases the risk for mortality in the general population (82) and in patients with CKD not on dialysis (10, 34–36, 38). In hemodialysis patients, hypernatremia was observed to be a risk factor for all-cause mortality (44) and mortality risk for causes other than cardiovascular disease or malignancy in a second study (46). Whether hypernatremia is associated with mortality in peritoneal dialysis patients has not been studied.

### Treatment of Dysnatremias in Patients With CKD Not on Dialysis

#### Treatment of Hyponatremia in Patients With CKD Not on Dialysis

**Figure 1:**
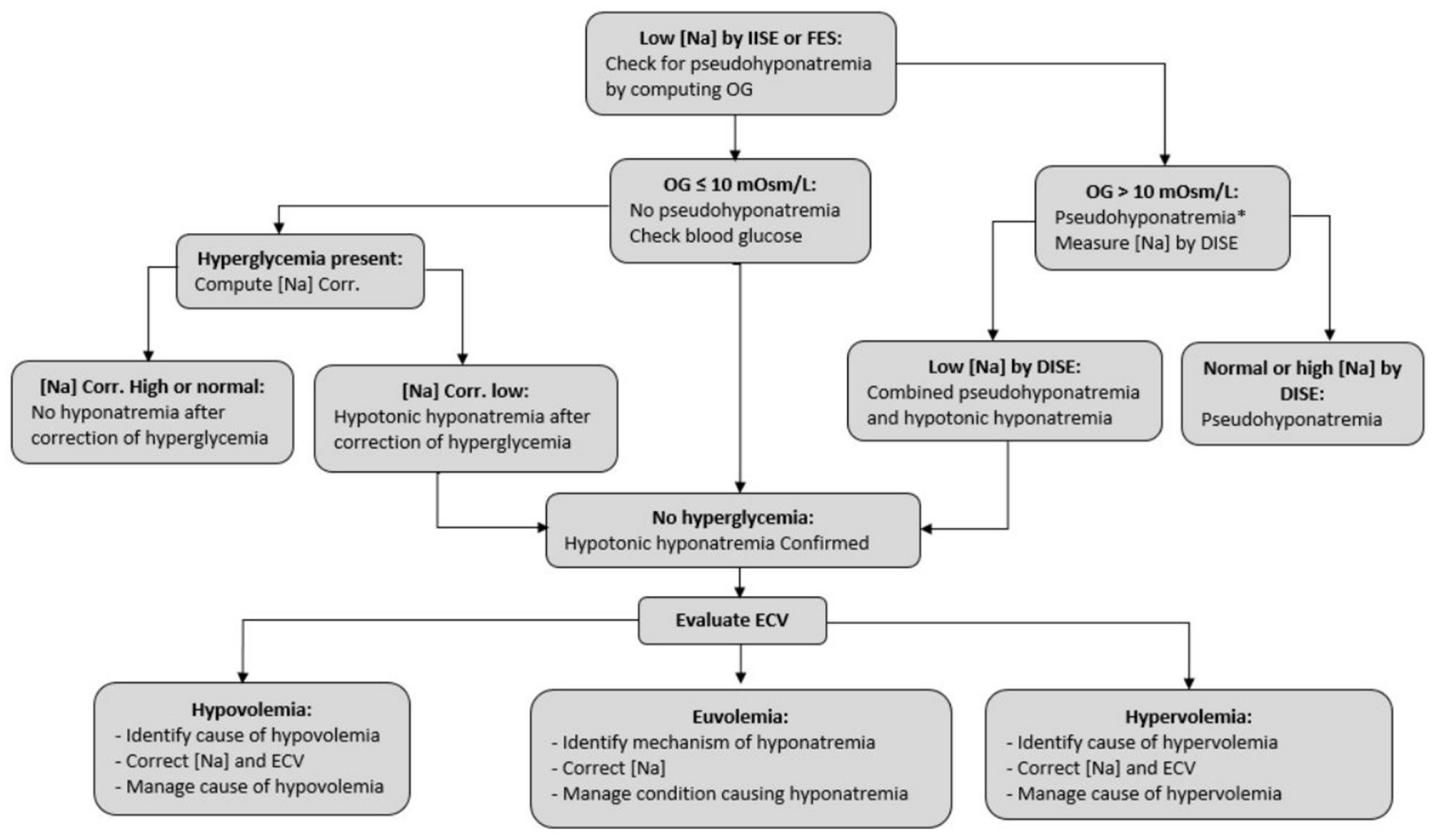
Steps in the management of hyponatremia. [Na], serum sodium concentration; IISE, indirect ion-specific electrode; FES, flame emission spectrophotometry; DISE, direct ion-specific electrode; OG, osmol gap, computed as measured serum osmolality minus the sum 2×[Na] + serum glucose + serum urea, where both glucose and urea concentrations are in mmol/L; ECV, extracellular volume. *Hyponatremia combined with a wide osmol gap and usually high serum osmolality can also be encountered when there is an excess of an exogenous solute with extracellular distribution, e.g., mannitol. Diagnosis of this case of hypertonic hyponatremia is obtained by history.

Guidelines (84, 85) and other reports (5, 19, 86, 87) address treatment of hyponatremia in the general population. Figure 1, based on Musso and Bargman's report (88), presents the steps of management of hyponatremia in all patients, such as those with CKD. The last step, evaluation of extracellular volume (ECV) is critical in determining the mechanism and guiding the management of hyponatremia in the general population (5, 19, 86, 87) and all categories of patients with CKD.

Table 3 summarizes the approaches to the treatment of hyponatremia in the general population (5). Infusion of hypertonic saline and restriction of fluid intake represent key general measures for treating hyponatremia in all patients with CKD, such as patients with anuria, while administration of loop diuretics or oral urea are effective means only in patients with significant renal function. Oral urea administration as the sole treatment of hyponatremia is safe and effective (89). Urea administration produces osmotic diuresis and increased free water clearance in the general population (90). Whether urea administration is also safe and effective in the early stages of CKD is not known currently. Even mild hyponatremia should be treated. Rondon-Berrios and Berl (91) proposed treating mild chronic hyponatremia with water intake limitation based on the electrolyte-free water clearance and oral administration of compounds increasing urinary water excretion such as sodium salts, urea, loop diuretics, and vasopressin inhibitors.

**Table 3:** Methods of treating hyponatremia in the general population (5).

**General measures**
-Hypertonic saline infusion (severe or moderate hyponatremia)
-Restriction of fluid intake (primary treatment in CKD-associated hyponatremia)
-Loop diuretics
-Urea administration
**Specific measures**
-Vaptans (hyponatremia associated with concentrated urine and high serum vasopressin levels)
-Desmopressin infusion (anticipated large water diuresis during treatment of hyponatremia caused by hypovolemia, primary polydipsia, beer potomania, and low
solute intake)
-Isotonic saline infusion (hypovolemic hyponatremia)
-Potassium salt administration (hyponatremia due to potassium deficit)
-Glucocorticoid administration (hyponatremia caused by cortisol deficit)
-Thyroid hormone replacement (hyponatremia caused by severe hypothyroidism)
-Increased dietary solute intake (hyponatremia due to low solute intake or beer potomania)
-Dialytic methods (hyponatremia in patients with advanced CKD)

We will address the correction of hyponatremia by saline infusion. In patients with CKD, the importance of an accurate estimate of TBW—in the calculation of the volume of infused saline, as well as in the overall management of these patients, acquires even greater importance than in patients with preserved renal function.

The aims of correcting hyponatremia by saline infusion include prevention of brain herniation, improvement of symptoms, and prevention of osmotic demyelination (5). The risk of osmotic demyelination from the rapid rise in [Na] is higher in chronic than in acute hyponatremia. During the rapid correction of chronic hyponatremia, organic osmolyte accumulation in the intracellular compartment of brain cells occurs slowly leading to shrinkage of their intracellular volume. These changes are thought to be linked to the development of osmotic myelinolysis (92). The European guidelines recommend rapid correction of hyponatremia with severe symptoms, regardless of whether the hyponatremia is acute or chronic, by the infusion of hypertonic saline, frequent measurements of [Na] in the first hour of treatment, and evaluation of the symptoms after the increase of [Na] by 5 mmol/L; if symptoms improve, the guidelines recommend stopping the hypertonic saline and limiting the rise in [Na] to a total of 10 mmol/L within the first 24 h and then managing the condition that caused the hyponatremia and increasing [Na] by 8 mmol/L every 24 h thereafter until [Na] reaches 130 mmol/L: if symptoms do not improve, the guidelines suggest continuing the infusion of hypertonic saline with an increase in [Na] by 1 mmol/L hourly until a total rise of 10 mmol/L or [Na] reaches 130 mmol/L (85). Tandukar et al. (93) summarized the findings of 19 publications reporting 21 patients who developed osmotic demyelination syndrome after the correction of hyponatremia by ≤10 mmol/L per 24 h. Sterns and coauthors recommend a change in [Na] during treatment of symptomatic chronic hyponatremia up to 6 mmol/L in the first 6 h, unless symptoms persist when even higher rates of increase in [Na] are indicated, 6–8 mmol/L in the first 24 h, 12–14 mmol/L in 48 h, and 14–16 mmol/L in 72 h (94). Overcorrection should be prevented rigorously (95) and corrected promptly with hypotonic infusions if it occurs. Acute hyponatremia may be treated faster. Whether the same rates of correction of hyponatremia should be applied to patients with early stages of CKD and those treated by dialysis will need to be investigated. In the absence of new information, it would be prudent to apply these guidelines along with clinical judgment to patients with hyponatremic CKD.

Changes in [Na] resulting from the change in body sodium content are determined by the total change in body sodium and by TBW (6). Consequently, TBW, which changes in the same direction and by the same magnitude as ECV in isotonic ECV disturbances, is used when calculating the volume of saline-infused to correct hyponatremia. The Adrogué–Madias formula, which has been used extensively in the management of hyponatremias, calculates the rise in [Na] after infusion of 1 L of saline hypertonic to the serum as follows (86):

(2) $$\begin{array}{r} {\left[Na\right]}_{Final}-{\left[Na\right]}_{Initial}=\frac{{\left[Na\right]}_{Infusate}-{\left[Na\right]}_{Initial}}{TBW_{Initial}+1} \end{array}$$

Where [Na]_Initial_ is the pre-infusion and [Na]_Final_ is the post-infusion [Na], [Na]_Infusate_ is the sodium concentration in the infused saline, and TBW_Initial_ is the pre-infusion TBW. Determination of the volume of infused saline (V_Infused_) required to produce the desired rise in [Na] using this formula requires one more calculation. For example, in a patient with [Na]_Initial_ = 110 mmol/L and TBW_Initial_ = 40 L, the Adrogué–Madias formula calculates that infusion of 1 L of 513 mmol/L (3%) saline will result in a 9.8 mmol/L rise in [Na]. If the desired rise in [Na] is 6 mmol/L, V_Infused_ = 1 × 6/9.8 = 0.6 L. A formula published subsequently, and based on the same principles as the Adrogué–Madias formula, calculates directly V_Infused_, as follows (19):

(3) $$\begin{array}{r} V_{Infused}=TBW_{Initial}\times \frac{{\left[Na\right]}_{Final}-{\left[Na\right]}_{Initial}}{{\left[Na\right]}_{Infusate}-{\left[Na\right]}_{Final}} \end{array}$$

In the above example, Equation(3) calculates also a V_Infused_ of 0.6 L.

One potential source of variance between [Na]_Final_ values predicted by Equations (2) or (3) and observed relates to the fact that these formulas do not include urinary excretions of water, sodium, and potassium. The urinary excretion may vary considerably during treatment between various categories of hyponatremia (19). Renal excretion of water and monovalent cations during treatment may be less of an issue in patients with advanced CKD.

Another source of variance may be due to an inaccurate estimate of TBW_Initial_ (19). Hyponatremias in both the general and CKD populations can develop in the settings of hypovolemia, euvolemia, or hypervolemia (5, 19). Several conditions and pathogenic mechanisms are responsible for the development of hyponatremia in patients with normal, low, or high ECV, and consequently abnormal TBW_Initial_ values. The importance of the estimate of TBW_Initial_ entered in formulas 2 or 3 is greater in patients with CKD, because of the limited capacity of their kidney to correct errors. Whether ECV and TBW are normal, i.e., whether patients are at their dry weight, is critical in advanced CKD not only for the evaluation of the mechanism and for the management of dysnatremias, but also other clinical reasons, e.g., prevention of cardiovascular complications of hypervolemia or management of hypertension.

Several methods have been proposed for estimating TBW in hyponatremic patients. The older method calculates TBW as an arbitrary fraction of body weight, the difference between women and men, and the difference between older and younger individuals in each gender (86). This method may provide estimates with error because it does not account for body composition at dry weight, in particular about the body fat content, or changes in TBW accompanying the hyponatremia. In addition, the use of sex differences poses a challenge among transgender patients.

Another method for estimating TBW is based on the anthropometric formulas, which represent statistical regression analyses in populations with euvolemia. These statistical analyses compared the TBW measurements by isotopic dilution methods and gender, age, body weight, height, and, in some formulas, the race of the studied subjects. The formulas provided by these studies appropriately account for weight change secondary to a change in body fat, but not for weight change caused by a change in body water. Water gain increases body weight and the fraction of TBW/body weight, while the gain in body fat increases body weight and TBW but decreases the fraction of TBW/body weight (96). The anthropometric formulas always calculate a decrease in the fraction TBW/body weight as weight increases (96). In patients with disorders of TBW, TBW should be estimated as the TBW at dry weight calculated by an anthropometric formula plus or minus the difference between actual and dry weights (19, 96, 97). The drawbacks of this method are that anthropometric formulas, which have large margins of error, and that dry weight is not known in many cases and even when known it may have been miscalculated.

Various other methods, which have been applied for evaluating ECV and TBW in dialysis patients, have limitations (98, 99). ECV associated with optimal perfusion of vital organs, i.e., with the effective arterial blood volume, may vary from the normal value in disease states, e.g., in congestive heart failure (100). Measurement of TBW and ECV by bioimpedance and simultaneous performance of lung ultrasonography to evaluate the extravascular lung ECV is considered a promising method for estimating the dry weight (98, 101, 102). Careful clinical evaluation of the fluid status of patients on hemodialysis is imperative (103, 104). In the future, bioimpedance may become the method of choice for the TBW value used to calculate the volume of the saline infusate in hyponatremic CKD patients. The management of ECV in these patients is best done by combining clinical evaluation, bioimpedance, and lung ultrasonography.

Another source of imprecision in Equations 2 and 3 is that they do not account for exchanges of sodium between the extracellular compartment and the osmotically inactive sodium stored in polyanionic proteoglycans, mainly glycosaminoglycan found in skin, cartilage, and other tissues when [Na] is changing (7). In a study of infusion of hypertonic saline in normal individuals, the rise in [Na] at the end of infusion was almost identical to the rise predicted by the Adrogué–Madias formula, but [Na] decreased 4 h later (105); this decrease in [Na] was not explained by the urinary losses of sodium, potassium, and water. The authors of this study proposed that it was due to the uptake of sodium by proteoglycans. For all these reasons, monitoring of [Na] during and after saline infusion for correction of hyponatremia is critical in patients with or without CKD (19). Measuring urine volume and monovalent cation concentrations in urine will not be needed in every case, but will be useful in some cases with early stages of CKD, foremost in cases of hypovolemic hyponatremia in which infusion of hypertonic saline not only will raise [Na] but also at some point will remove the volume stimulus for vasopressin release, which will result in the production of large volumes of dilute urine. Monitoring urine volume and measuring sodium and potassium concentrations in this urine volume when treatment started and after urine volume starts increasing may provide important therapeutic information.

#### Treatment of Hypernatremia in Patients With CKD Not on Dialysis

Hypernatremia may also develop in the setting of hypovolemia, euvolemia, or hypervolemia (82, 106). The proper management of hypernatremia requires the identification of the underlying cause and careful correction (107). The prevention of cerebral edema during treatment is of paramount importance. If hypernatremia is chronic (≥48 h) or of unknown duration, correction of [Na] should not exceed 0.5–1.0 mmol/L per hour or 8–10 mmol/L in the first 24 h, to prevent cerebral edema, permanent neurologic damage, or death (107). Asymptomatic chronic hypernatremia may be corrected over 48 h or longer. More rapid correction (up to 1 mmol/L per hour, up to 8–15 mmol/L over the first 8 h) may be appropriate if the onset of hypernatremia is acute (<48 h), e.g., in accidental sodium loading, and unstable patients (107). In these settings, rapid correction improves the prognosis without increasing the risk of cerebral edema (107). The volume of hypotonic saline needed to produce the desired decrease in [Na] can be computed by modification of Equation 3 as follows (82):

(4) $$\begin{array}{r} V_{Infused}=TBW_{Initial}\times \frac{{\left[Na\right]}_{Initial}-{\left[Na\right]}_{Final}}{{\left[Na\right]}_{Final}-{\left[Na\right]}_{Infusate}} \end{array}$$

Hypernatremia can be corrected by infusion intravenously of sterile water (108). When infusion of sterile water is used, Equation 4 should be modified as follows (82):

(5) $$\begin{array}{r} V_{Infused}=TBW_{Initial}\times \frac{{\left[Na\right]}_{Initial}-{\left[Na\right]}_{Final}}{{\left[Na\right]}_{Final}} \end{array}$$

Monitoring of [Na] is also imperative during treatment and for a period of at least 24 h after the treatment of hypernatremia with the infusion of hypotonic fluid.

#### Treatment of Dysnatremias by Dialytic Methods

Dangoisse et al. (109) and Rosner and Connor (110) summarized the principles of management of dysnatremias by continuous renal replacement therapy (CRRT): this management requires customizing either the CRRT circuit or the dialysis solution. The CRRT circuit in continuous venovenous hemofiltration or hemodiafiltration can be customized by computing the sodium concentration at the end of the circuit and then adding a post-filter replacement volume of hypertonic or hypotonic fluid calculated to bring the sodium concentration of the circuit to the desired level. The CRRT dialysis solutions for treating severe dysnatremias are customized by adding to the dialysis solution an appropriate volume of hypertonic saline for hypernatremia or sterile water for hyponatremia calculated by a nomogram to bring the sodium concentration of the dialysis fluid to the desired level. The drawbacks of this approach are that the concentrations of other important ingredients of the dialysis fluid, e.g., potassium, calcium, and magnesium are decreased and there are increased chances of error (110).

Dysnatremias are treated by hemodialysis by changing the sodium concentration of the dialysis fluid. The treatment of dysnatremias by peritoneal dialysis will be presented in the peritoneal dialysis section. Attention is required for the prevention of errors during the treatment of severe dysnatremia by dialytic methods (110–113). These procedures require performance in intensive care units by well-trained personnel. During the procedure, the patient should be clinically monitored, and [Na] should be determined frequently, e.g., every 1–2 h. Clinical monitoring should continue and blood chemistries and concentration of sodium in the dialysis fluid should be repeated after the procedure. Point-of-care chemistry measurements are required. Careful application of multidisciplinary protocols for managing dysnatremias by dialytic methods may improve patient outcomes (114).

#### Treatment of Hyponatremia by Dialytic Methods

Dialytic methods employed in the treatment of hyponatremia include CRRT methods, intermittent hemodialysis, and peritoneal dialysis. Table 4 contains reports of management of hyponatremia by dialytic methods (115–130). One issue that requires further study in hyponatremia treated by hemodialysis is the effect of a decrease in plasma urea concentration during the procedure. Experimental studies have demonstrated that a urea concentration gradient between the brain intracellular and extracellular compartments at the end of a hemodialysis session causes osmotic entry of fluid into the brain cells and can contribute to the dialysis disequilibrium syndrome: following hemodialysis, urea concentration in the brain cells of azotemic rats was higher than the corresponding plasma concentration and the brain cells exhibited water gain (131). In addition, the accumulation of organic osmolytes, in particular myoinositol and taurine, was higher in brain cells of azotemic than non-azotemic rats 24 h after correction of chronic hyponatremia (132).

**Table 4:** Treatment of hyponatremia by dialytic methods.

**Dialytic method**	**Reference study**
**CRRT**	
CVVH	Ji et al. (115)
	Ostermann et al. (116)
	Yessayan et al. (117)
	Algurashi et al. (118)
CVVHD	Bender (119)
	Vassalo et al. (120)
	Victorsdottir et al. (121)
CVVHDF	Tandukar et al. (122)
**Intermittent hemodialysis**	
	Wendland et al. (123)
	Lew et al. (124)
	Courteau et al. (125)
	Kodama et al. (126)
	Lew et al. (127)
**Peritoneal dialysis**	
	Gundy and Trafford (128)
	Berger et al. (129)
	Inagaki et al. (130)

*CRRT, continuous renal replacement therapy; CVVH, Continuous veno-venous hemofiltration; CVVHD, continuous veno-venous hemodialysis; CVVHDF, continuous veno-venous hemodiafiltration*.

On the basis of rapid correction of severe hyponatremia by hemodialysis with no adverse consequences in one patient, it was suggested that azotemia protects from such consequences when hyponatremia is treated by hemodialysis (133). However, osmotic demyelination syndrome developed after the correction of [Na] from 100 to 121 mmol/L over 2.5 h during the first hemodialysis of a patient with pre-dialysis blood urea of 36.4 mmol/L or 218 mg/dl (134). Sirota and Berl (135) suggested that the risk of osmotic demyelination from rapid correction of hyponatremia by hemodialysis may be low in patients who have been on a regular chronic hemodialysis schedule but is substantially higher in patients starting hemodialysis. The treatment of hyponatremia by hemodialysis using the correction rates of [Na] recommended by existing guidelines for the general population should be combined with a slow rate of reduction of blood urea level by the use of shorter dialysis times, lower blood or dialysis fluid flow rates, less efficient dialyzers, or combinations of these measures. More frequent hemodialysis sessions can compensate for the slow removal of azotemic substances.

The methodology used for treating hyponatremia by peritoneal dialysis requires either the addition to the peritoneal dialysis solution of a volume of hypotonic saline calculated to bring the sodium concentration of this solution to the desired level (130), or performance of hourly exchanges of dialysis solution containing 4.25% dextrose (9). In this last method, free water transfers from the blood compartment to the peritoneal cavity during the early phase of a peritoneal dialysis exchange when using hypertonic dialysis fluid because of sodium sieving (136). One azotemic patient who was treated for the first time with peritoneal dialysis for a [Na] of profound 101 mmol/L developed osmotic myelinolysis with a fatal outcome (137). [Na] of this patient was 126 mmol/L on day 2 and 138 mmol/L on day 3.

#### Treatment of Hypernatremia by Dialytic Methods

Dialytic methods for treating hypernatremia include CRRT (138–142), intermittent hemodialysis (124, 143, 144), and peritoneal dialysis (25, 130, 145). Management of hypernatremia by CRRT is done by customizing the dialysis fluid (139, 141, 142), or infusing hypertonic sodium solutions when there is an urgent need for the very slow rate of reduction in [Na], e.g., in patients with hypernatremia and cerebral edema (140). The rate of reduction of [Na] in hypernatremic patients treated with CRRT should be monitored. In a retrospective study, the hourly rate of reduction in [Na] > 1 mmol/L and dependency on vasopressors were shown to be risk factors for mortality (146). In contrast, a prospective study found no relation between the rate of change in [Na] during CRRT and mortality in dysnatremic patients (147).

Hypernatremia is corrected by hemodialysis by modifying the sodium concentration in the hemodialysis fluid (124, 127, 144). The rate of correction of hypernatremia should be the same as in the section of treatment in patients with CKD not on dialysis. For this reason, the sodium concentration in the dialysis fluid should not differ from [Na] greatly and dialysis should be slow as detailed in the section on the treatment of hyponatremia by hemodialysis. A proposed novel method of producing dialysis fluid allows a larger range of sodium concentration in the dialysis fluid without altering the concentrations of other important ingredients, e.g., potassium and calcium, in this fluid (127). Correction of hypernatremia by peritoneal dialysis is done by lowering the sodium concentration of the dialysis fluid (25) or by using the commercial dialysis solution in documented acute cases in which hypernatremia can be treated at a fast rate (145).

### Needs for Future Studies

There are important deficits in our knowledge about the treatment of dysnatremias in patients with CKD not on dialysis and by dialytic methods, particularly by peritoneal dialysis. These deficits include the targets of change in [Na], the best method to achieve these targets, the problems encountered during treatment, and particularly the outcomes of the treatments (113, 148). Whether treatment of dysnatremias in CKD improves mortality is another important question that needs to be studied.

Finally, the prevention of dysnatremias is an important factor in the management of patients with CKD. The range of fluid intake that allows maintenance of [Na] within the normal range in this patient group remains limited. Careful instruction to all patients about fluid intake and identification of patients who have experienced or may be prone to dysnatremias because of dietary habits, associated diseases, or medications affecting thirst, should be part of the routine management of patients with CKD. A recent review of thirst in hemodialysis patients identified differences between these patients and individuals with normal kidney function and areas where our curiosity remains unfulfilled (149). Studies of thirst in patients with CKD may constitute a critical step in the prevention of dysnatremias.

## Conclusions

Dysnatremias occur frequently in patients with CKD and are associated with adverse outcomes. The aim of the treatment of dysnatremias is the same in patients with CKD and the general population. Whether azotemia permits faster rates of correction of dysnatremias in patients with CKD and whether treatment of these conditions by renal replacement methods improves patient outcomes are questions that need to be studied. Prevention of dysnatremias in patients with CKD requires careful patient education about fluid intake and further research on thirst regulation and on conditions affecting thirst in this patient population.

## Author Contributions

SA and MU: conceptualization. SA, SL, TI, AT, and MU: literature review. SA, AT, and MU: methodology. SA and AT: writing – original draft preparation. SL, TI, and MU: writing – review and editing. All the authors contributed to the article and approved the submitted version.

## Funding

This study has been supported and funded by Dialysis Clinics Inc., (DCI). Grant ID: 3RGX8-FP00007518. IRB ID: 19-429.

## Conflict of Interest

The authors declare that the research was conducted in the absence of any commercial or financial relationships that could be construed as a potential conflict of interest.

## Publisher's Note

All claims expressed in this article are solely those of the authors and do not necessarily represent those of their affiliated organizations, or those of the publisher, the editors and the reviewers. Any product that may be evaluated in this article, or claim that may be made by its manufacturer, is not guaranteed or endorsed by the publisher.
